# A 3D and Explainable Artificial Intelligence Model for Evaluation of Chronic Otitis Media Based on Temporal Bone Computed Tomography: Model Development, Validation, and Clinical Application

**DOI:** 10.2196/51706

**Published:** 2024-08-08

**Authors:** Binjun Chen, Yike Li, Yu Sun, Haojie Sun, Yanmei Wang, Jihan Lyu, Jiajie Guo, Shunxing Bao, Yushu Cheng, Xun Niu, Lian Yang, Jianghong Xu, Juanmei Yang, Yibo Huang, Fanglu Chi, Bo Liang, Dongdong Ren

**Affiliations:** 1 ENT Institute and Department of Otorhinolaryngology Eye & ENT Hospital Fudan University Shanghai China; 2 NHC Key Laboratory of Hearing Medicine Research Eye & ENT Hospital Fudan University Shanghai China; 3 Department of Otolaryngology—Head and Neck Surgery Vanderbilt University Medical Center Nashville, TN United States; 4 Department of Otorhinolargnology Union Hospital, Tongji Medical College Huazhong University of Science and Technology Wuhan China; 5 State Key Laboratory of Digital Manufacturing Equipment and Technology School of Mechanical Science and Engineering Huazhong University of Science and Technology Wuhan China; 6 Department of Electrical and Computer Engineering Vanderbilt University Nashville, TN United States; 7 Department of Radiology Eye & ENT Hospital Fudan University Shanghai China; 8 Department of Radiology Union Hospital, Tongji Medical College Huazhong University of Science and Technology Wuhan China

**Keywords:** artificial intelligence, cholesteatoma, deep learning, otitis media, tomography, x-ray computed, machine learning, mastoidectomy, convolutional neural networks, temporal bone

## Abstract

**Background:**

Temporal bone computed tomography (CT) helps diagnose chronic otitis media (COM). However, its interpretation requires training and expertise. Artificial intelligence (AI) can help clinicians evaluate COM through CT scans, but existing models lack transparency and may not fully leverage multidimensional diagnostic information.

**Objective:**

We aimed to develop an explainable AI system based on 3D convolutional neural networks (CNNs) for automatic CT-based evaluation of COM.

**Methods:**

Temporal bone CT scans were retrospectively obtained from patients operated for COM between December 2015 and July 2021 at 2 independent institutes. A region of interest encompassing the middle ear was automatically segmented, and 3D CNNs were subsequently trained to identify pathological ears and cholesteatoma. An ablation study was performed to refine model architecture. Benchmark tests were conducted against a baseline 2D model and 7 clinical experts. Model performance was measured through cross-validation and external validation. Heat maps, generated using Gradient-Weighted Class Activation Mapping, were used to highlight critical decision-making regions. Finally, the AI system was assessed with a prospective cohort to aid clinicians in preoperative COM assessment.

**Results:**

Internal and external data sets contained 1661 and 108 patients (3153 and 211 eligible ears), respectively. The 3D model exhibited decent performance with mean areas under the receiver operating characteristic curves of 0.96 (SD 0.01) and 0.93 (SD 0.01), and mean accuracies of 0.878 (SD 0.017) and 0.843 (SD 0.015), respectively, for detecting pathological ears on the 2 data sets. Similar outcomes were observed for cholesteatoma identification (mean area under the receiver operating characteristic curve 0.85, SD 0.03 and 0.83, SD 0.05; mean accuracies 0.783, SD 0.04 and 0.813, SD 0.033, respectively). The proposed 3D model achieved a commendable balance between performance and network size relative to alternative models. It significantly outperformed the 2D approach in detecting COM (*P*≤.05) and exhibited a substantial gain in identifying cholesteatoma (*P*<.001). The model also demonstrated superior diagnostic capabilities over resident fellows and the attending otologist (*P*<.05), rivaling all senior clinicians in both tasks. The generated heat maps properly highlighted the middle ear and mastoid regions, aligning with human knowledge in interpreting temporal bone CT. The resulting AI system achieved an accuracy of 81.8% in generating preoperative diagnoses for 121 patients and contributed to clinical decision-making in 90.1% cases.

**Conclusions:**

We present a 3D CNN model trained to detect pathological changes and identify cholesteatoma via temporal bone CT scans. In both tasks, this model significantly outperforms the baseline 2D approach, achieving levels comparable with or surpassing those of human experts. The model also exhibits decent generalizability and enhanced comprehensibility. This AI system facilitates automatic COM assessment and shows promising viability in real-world clinical settings. These findings underscore AI’s potential as a valuable aid for clinicians in COM evaluation.

**Trial Registration:**

Chinese Clinical Trial Registry ChiCTR2000036300; https://www.chictr.org.cn/showprojEN.html?proj=58685

## Introduction

Chronic otitis media (COM) represents a recurrent inflammatory condition inside the tympanic cavity [1]. COM encompasses various forms, including chronic suppurative otitis media (CSOM) and cholesteatoma, each with unique histological characteristics. CSOM involves the accumulation and discharge of purulent fluid, affecting an estimated 330 million people worldwide, with approximately half experiencing hearing loss [2]. Cholesteatoma is characterized by the buildup of keratinized squamous epithelium, which has the potential to erode auditory structures and exhibits a notable tendency for relapse. Accurate identification and differentiation of COM types are crucial for effective disease management and surgical planning [3]. Mastoidectomy, which involves the removal of part of the temporal bone, is the conventional surgical approach for COM. However, less invasive techniques such as endoscopic tympanoplasty are gaining favor for treating CSOM and other noncholesteatoma conditions due to their potential for reduced structural damage and faster recovery [4-9].

Temporal bone computed tomography (CT) is vital for assessing COM and aiding in surgical planning, especially when initial otoscopic examinations have restricted views and yield inconclusive findings [10]. Offering a cost-effective alternative to magnetic resonance imaging (MRI), CT is instrumental in distinguishing cholesteatoma from CSOM by detecting osseous erosion in the tympanum. Although studies have shown that clinicians are capable of diagnosing COM based on CT alone [11-17], distinguishing between COM subtypes poses greater challenges to the human eye. Moreover, interpreting temporal bone CT scans requires specialized training and experience, which may not be universally available across otolaryngologists.

Artificial intelligence (AI) is making remarkable advancements in health care. Deep learning (DL) models, particularly convolutional neural networks (CNNs), have demonstrated enhanced efficiency and reduced errors in disease diagnoses and prediction of clinical outcomes [18-21]. While a few recent papers have reported CNN models in evaluating COM with accuracy scores ranging from 0.77 to 0.85, these studies primarily relied on otoscopic or single-layer CT scans [22,23]. These 2D representations may not be optimal for revealing pathological changes in concealed or peripheral anatomical structures, such as the attic space and the mastoid air cells. In addition, the inherent “black box” nature of DL models, where decision-making strategies are challenging to understand, has been a common criticism [24,25]. This lack of comprehensibility hinders the widespread adoption of AI models in clinical practice.

In light of these challenges, this study aimed to create an explainable, 3D CNN model for the automatic interpretation of temporal bone CT scans. The model was designed to pinpoint the region of interest (ROI) and identify pathological and cholesteatomatous conditions in a 3D fashion. Comprehensive benchmarks against baseline methods and human experts on distinct data sets were conducted to demonstrate the robustness and generalizability of this model. In addition, heat map generation was used to highlight potential pathological changes in CT scans and elucidate the model’s rationale for making predictions. These features were integrated into an AI system for the automatic, end-to-end evaluation of COM, which was subsequently assessed in clinical settings. The overarching goal of this system is to support clinicians in making informed decisions for common otologic conditions, thereby enhancing efficiency, reliability, and transparency.

## Methods

### Ethical Considerations

This study was conducted in accordance with the principles of the Declaration of Helsinki. Ethical approval was granted by the institutional review boards at Vanderbilt University Medical Center (191804) and the Eye, Ear, Nose and Throat (EENT) Hospital of Fudan University (2019076). Informed consent was waived as all data were de-identified. The observational study, which aimed to assess the model’s viability in aiding preoperative assessment, was registered with the Chinese Clinical Trial Register (ChiCTR: 2000036300). No compensation was provided to any study participants.

### Participants

**Figure 1:**
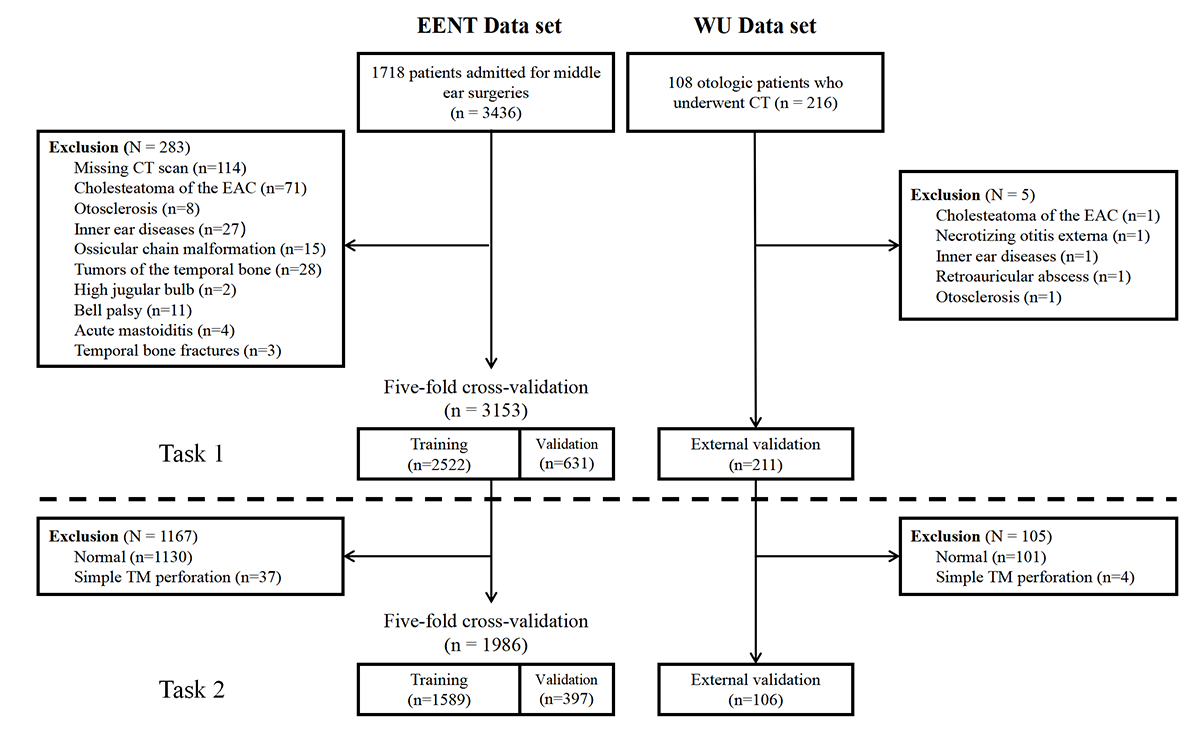
Flowchart of data retrieval. CT: computed tomography; EAC: external auditory canal; EENT: Eye, Ear, Nose, and Throat Hospital of Fudan University; TM: tympanic membrane; WU: Wuhan Union Hospital.

Data were retrospectively obtained from patients admitted for middle ear surgeries from December 2015 to July 2021 at EENT Hospital. Patients diagnosed with acute otitis media, any inner or external ear diseases, or those with missing temporal bone CT scan were excluded, resulting in 1661 patients eligible for model development. An extra data set containing 108 patients with COM was collected from Wuhan Union (WU) Hospital for external validation (Figure 1).

### Temporal Bone CT Scans

As part of the routine preoperative assessment, each patient underwent at least 1 temporal bone CT, conducted from the lower margin of the external auditory meatus to the top margin of the petrous bone using a SOMATOM Sensation 10 CT scanner (Siemens Inc) at the EENT Hospital. The scanning parameters were as follows: matrix (512 × 512), field of view (220 mm × 220 mm), tube voltage (140 kV), tube current (100 mAs), section thickness (0.6-0.75 mm), window width (4000 HU), and window level (700 HU). CT scans from the WU Hospital were obtained using a SOMATOM Plus 4 model (Siemens Inc) with different settings for field of view (100 mm), voltage (120 kV), and thickness (0.75 mm). All images were saved in the DICOM format.

### Label Assignment

All eligible ears were treated as independent cases and assigned ground truth labels based on their diagnoses (Table 1). Each label was verified according to intraoperative findings and pathology reports for operated ears and using a combination of history, ear examination, audiogram results, and imaging findings for unoperated ears. In cases of unoperated ears, a “normal” label was assigned when there was an absence of ear symptoms, hearing loss, or signs of inflammation. A diagnosis of CSOM was assigned when chronic purulent discharge, conductive hearing loss, and the presence of a perforated tympanic membrane or soft tissue shadow in the tympanic cavity were observed. Cholesteatoma was considered if keratin debris was identified, or if there were signs of osseous damage along with retraction or perforation of the pars flaccida [22]. Two otolaryngology residents with full access to patients’ medical records independently reviewed these labels as unblinded annotators. Any discrepancies were addressed with senior specialists until a consensus was reached. All data were deidentified and stored on password-protected computers.

**Table 1:** Summary of patient characteristics and label assignment.

Characteristics				EENT^a^ data set (N=1661; number of ears=3153)		WU^b^ data set (N=108; N=211)
Patient age (years), mean (SD)				41.1 (16.6)		39.8 (14.0)
**Patient sex,** **n (%)**						
	Male			832 (50.1)		49 (45.4)
	Female			829 (49.9)		59 (54.6)
**Diagnosis per ear, n (%)**						
		Normal	1130 (35.8)		101 (47.9)	
		Cholesteatoma	728 (23.1)		30 (14.2)	
		CSOM^c^	1011 (32.1)		69 (32.7)	
		Tympanosclerosis	142 (4.5)		2 (0.1)	
		Cholesterol granuloma	72 (2.3)		1 (0.05)	
		OME^d^	41 (1.3)		7 (3.3)	
		Adhesive otitis media	29 (0.1)		1 (0.05)	
**Task 1 labels, n (%)**						
		Normal	1130 (35.8)		101 (47.9)	
		Pathological	2023 (64.2)		110 (52.1)	
**Task 2 labels, n (%)**						
		Cholesteatoma	728 (36.7)		28 (26.4)	
		Noncholesteatoma	1258 (63.3)		78 (73.6)	

^a^EENT: Eye, Ear, Nose, and Throat Hospital of Fudan University.

^b^WU: Wuhan Union Hospital.

^c^CSOM: chronic suppurative otitis media.

^d^OME: otitis media with effusion.

### Model Architecture

The framework consists of 2 functionally distinct units: a region proposal network for 3D segmentation of ROI, and a classification network for generating predictions. Both networks are established based on CNN models.

#### Region Proposal Network

**Figure 2:**
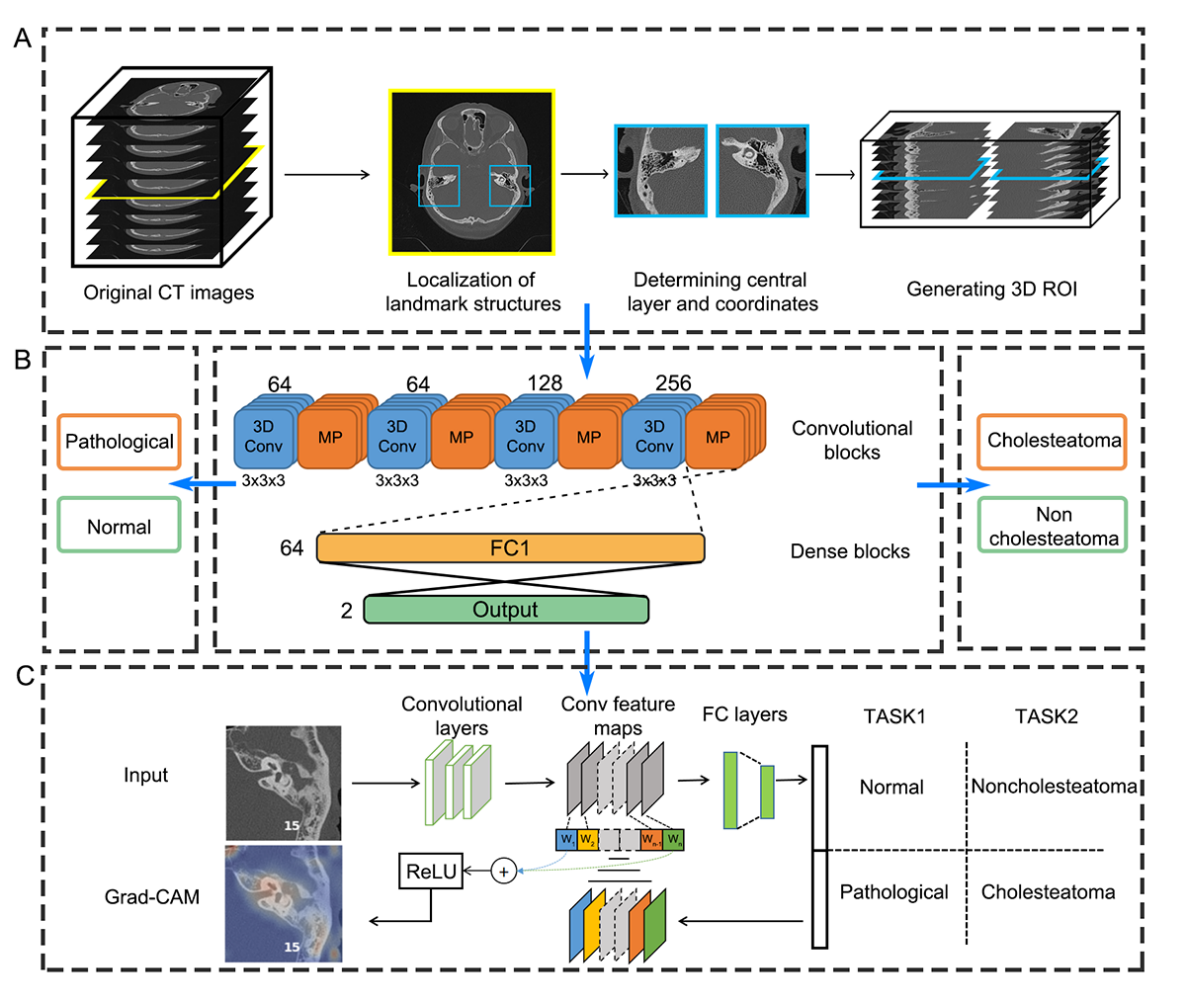
An overview of the AI framework. (A) The region proposal network used to locate landmark structures and segment the 3D ROI from the original CT scans. (B) The classification network based on a 3D convolutional neural network architecture and trained to perform 2 classification tasks. (C) The gradient heatmaps generated to highlight the critical regions for decision-making. Conv: convolution; CT: computed tomographic; FC: fully connected; MP: max pooling; ReLu: rectified linear unit; ROI: region of interest.

This network is designed to extract the middle ear on each side from a full set of temporal bone CT scan (Figure 2A). It contains a YOLO (You Only Look Once; v5) model that is trained to detect and locate 2 auditory structures, including the internal auditory canal and the horizontal semicircular canal, in a series of 2D axial CT scans [26]. These landmarks, positioned at or around the central level of the middle ear, possess unique graphical appearances recognizable by the object detection model. In our recent study, this model demonstrated a 100% success rate in identifying the middle ear region from temporal bone CT scans [22]. Subsequently, a 3D data matrix (150 × 150 × 32) of the ROI is extracted based on the center coordinates of these 2 structures on each side.

#### Classification Network

A 3D CNN model is built to interpret the extracted ROI and classify different types of conditions (Figure 2B). This model features 4 convolution blocks and 2 dense blocks (Table 2). Each convolution block consists of a 3D convolutional layer to summarize graphical features along all axes of the input image, followed by a max-pooling layer for downsampling these features and another layer for batch normalization. These high-level features are then pooled and passed to the fully connected layers of the dense blocks, where the diagnosis is predicted based on the calculated probability of each class by a softmax function. A dropout layer is applied to prevent overfitting [27].

**Table 2:** Architecture of the 3D convolutional neural network model.

Block and kernel inputs		Settings
**Convolution 1**		
	Conv3D^a^	(3,3,3,64)
	MaxPooling3D^b^	(2,2,2)
	BatchNormalization^c^	
**Convolution 2**		
	Conv3D	(3,3,3,64)
	MaxPooling3D	(2,2,2)
	BatchNormalization	
**Convolution 3**		
	Conv3D	(3,3,3,128)
	MaxPooling3D	(2,2,2)
	BatchNormalization	
**Convolution 4**		
	Conv3D	(3,3,3,256)
	MaxPooling3D	(2,2,2)
	BatchNormalization	
	GlobalAveragePooling3D^d^	
**Dense 1**		
	Fully connected	64
	Dropout	0.3
**Output**		
	Fully connected	2

^a^Conv3D: 3D convolutional layer.

^b^MaxPooling3D: 3D max pooling layer.

^c^BatchNormalization: batch normalization layer.

^d^GlobalAveragePooling3D: layer performing global average pooling for 3D data.

### Model Training and Testing

#### Task 1—Detection of COM

The first classification model was trained in a binary task distinguishing between normal and pathological ears in all cases (n=3153). The training and testing procedures involved 5-fold cross-validation on the internal (EENT) data set. Specifically, the data set was evenly partitioned into 5 nonoverlapping subsets in a random, stratified fashion. In each iteration, 1 subset was reserved for testing (n=631), while the remaining 4 were used for training (n=2522). Model performance metrics were averaged over 5 iterations of this process. During each training session, a random 20% of training images (n=504) were allocated for validation. Training was set for 1000 epochs with an initial learning rate of 0.0001, and the Adam optimizer was used to dynamically adjust the algorithm’s learning capability and minimize errors [28]. Early termination was implemented if no further decrease in validation loss was observed for a consecutive 10 epochs. These hyperparameters were determined based on the resultant model performance and training efficiency shown in a preliminary study. The trained model was also evaluated on the external data set (n=211) in each round.

#### Task 2—Identification of Cholesteatoma

The second classification model was trained to specifically identify cholesteatoma on selected CT scans that displayed signs of inflammation in the middle ears. This task was designed to simulate a common clinical scenario where clinicians need to differentiate cholesteatoma from other types of COM in patients with positive imaging findings. The aim was to provide a preoperative assessment of the risk of cholesteatoma, assisting clinicians in surgical planning [3,29]. For this task, a subset of CT scans with visible soft tissue density or increased opacification in the middle ear or mastoid was selected from both the internal (n=1986) and external sets (n=106). The remaining methods, including extraction of ROI, network architecture, and the training and testing procedures, were consistent with those used in the first task.

### Ablation Study

To refine model selection and gain a better understanding of the network’s behavior, an ablation study was performed to compare the proposed classification network with 3 alternative models, each incorporating modifications to certain features. Specifically, the number of convolutional blocks was decreased and increased by 1 in alternative model 1 and model 2, respectively, and a different size of filter was applied in model 3 (Tables S1-S3 in Multimedia Appendix 1). To ensure adequate statistical power for detecting differences across models, experiments were conducted on the main data set using the same methodology as outlined in the preceding sections.

### Benchmarking Against the 2D Approach

To investigate whether the use of 3D CT scans may enhance diagnostic performance, a benchmark study was designed to compare the proposed system with a baseline model using 2D images. This baseline model, previously established by our team, uses transfer learning on a pretrained Inception-V3 (Google LLC) model [22]. In this study, the base model of Inception-V3 was retained, and the final classification layer was customized with a binary output. Training and validation were conducted in the same manner as the 3D model, except that only a single CT scan at the central layer of the ROI was used as the input for the 2D model. All image-preprocessing techniques and hyperparameter settings remained consistent with those outlined in the previous study [22].

### Benchmarking Against Human Experts

Another benchmark test was performed against human experts to provide an additional unbiased evaluation of the proposed system. Seven human specialists with a broad range of qualifications were recruited to perform both tasks based on the same image data. The participants included 2 senior otologists, each with 12 years of clinical experience, 1 senior head and neck radiologist with 21 years of experience, 1 attending otologist with 7 years of experience, and 3 otolaryngology residents with 3, 3, and 2 years of experience, respectively. Each expert was provided only with the CT scans and instructed to make a task-specific diagnosis to each ear (task 1: normal or pathological; task 2: cholesteatoma or noncholesteatoma). The test data for clinicians comprised a random selection of 244 ears from the EENT set and all eligible ears from the WU set. To assess intrarater reliability, a random replication of 10% of test cases (n=48) was mixed with these data. All test cases (N=502) had not been previously seen by any experts. They were anonymized, shuffled, and stored on a password-protected computer along with spreadsheets to record each expert’s diagnoses for these cases.

### Generation of Heat Maps

Gradient-Weighted Class Activation Mapping was used to visualize model’s rationale for decision-making (Figure 2C). In essence, this approach leverages the gradients of the target class flowing into the final convolutional layer to produce a coarse localization heat map, highlighting the critical regions in the image [30]. In this study, heat maps were generated in a 3D fashion and rescaled to match the original images using TensorFlow 2.11 in Python 3.91 (Python Core Team) [31].

### Clinical Applications

The validated model was integrated into a Python program, enabling the automated assessment of COM from raw CT inputs to the generation of explainable diagnoses in an end-to-end fashion (see the section “Data Availability Statements” and Multimedia Appendix 2). To evaluate its viability in assisting otologists in clinical settings, this system was used with a prospective cohort of patients undergoing middle ear surgeries at EENT hospital from November 2023 to January 2024 in a single-arm observational study. Preoperative model predictions, along with routine assessments, were provided to 2 senior otologists, who were given autonomy to determine surgical strategies based on their discretion. Surgeons were surveyed regarding the use of model-generated information in their decision-making processes for these cases. Model predictions were used to analyze the selection of surgical approaches and to measure model performance against pathological findings. Hearing gain was assessed by comparing the air conduction threshold at 2 weeks postoperatively with the baseline.

### Statistical Analysis

Descriptive statistics were applied as appropriate. The overall predictability of a model was evaluated by the area under the receiver operating characteristic (AUROC) curve. The optimal cutoff threshold on the curve was determined at the point with minimal distance to the upper left corner on the validation set and subsequently applied to the test set. The numbers of correctly and incorrectly classified cases were displayed in a confusion matrix, and these were used to calculate the performance metrics, including accuracy, recall, specificity, precision, and *F*_1_-score. These metrics offer comprehensive insights into the model’s performance, covering overall correctness in identifying both positives and negatives (accuracy), sensitivity in detecting positive cases (recall), capability in ruling in patients (specificity), propensity for preventing false alarms (precision), and effectiveness in identifying positive cases while minimizing false positives and false negatives (*F*_1_-score). They were derived as shown in Textbox 1. Results are averaged over 5 iterations of cross-validation or external validation and presented as mean (SD). Intrarater consistency was evaluated using Cohen kappa. Significance was determined through pairwise 2-tailed *t* test for difference in performance between models and via 1-way analysis of variance between the proposed model and human experts. The alpha level was set at .05. Statistical analyses were conducted using Python 3.91 [31].

**The calculation of performance metrics.**

Accuracy = (True positive + True negative)/Total sample size

Recall = True positive/(True positive + False negative)

Specificity = True negative/(True negative + False positive)

Precision = True negative/(True negative + False negative)

*F*_1_-score=2 × True positive/(2 × True positive + False positive + False negative)

## Results

### ROI Extraction

**Figure 3:**
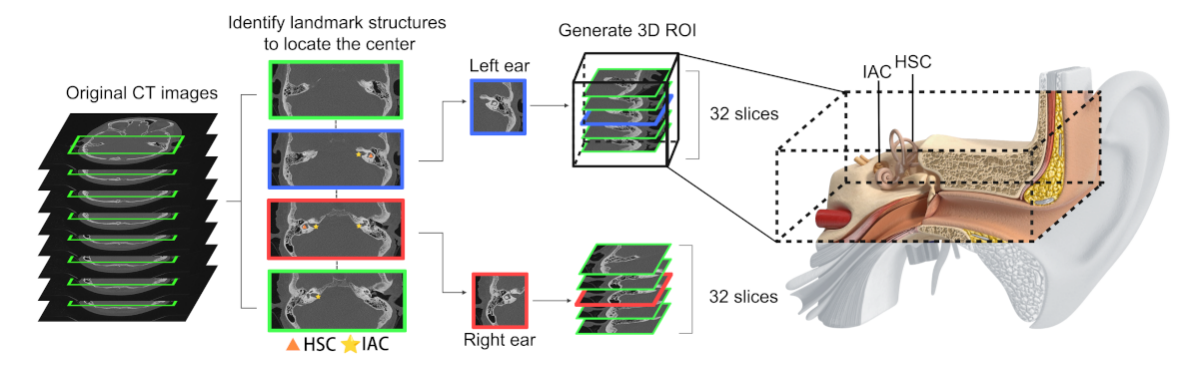
Generation of the 3D ROI. The region proposal network identifies landmark structures in each of the full-sized sequential CT slices and determines the center of the middle ear on each side. A 3D image comprising 32 stacks of axial slices in 150 × 150 pixels is subsequently segmented. This ROI encompasses an extensive range of critical anatomies within the temporal bone for the evaluation of COM. CT: computed tomographic; HSC: horizontal semicircular canal; IAC: internal auditory canal; ROI: region of interest.

The region proposal network successfully extracted the 3D ROI containing the critical anatomies on each side, including the tympanic cavity and sinus tympani (Figure 3). This has been confirmed by manual inspection of the generated images in all cases from both data sets.

### Task 1

**Figure 4:**
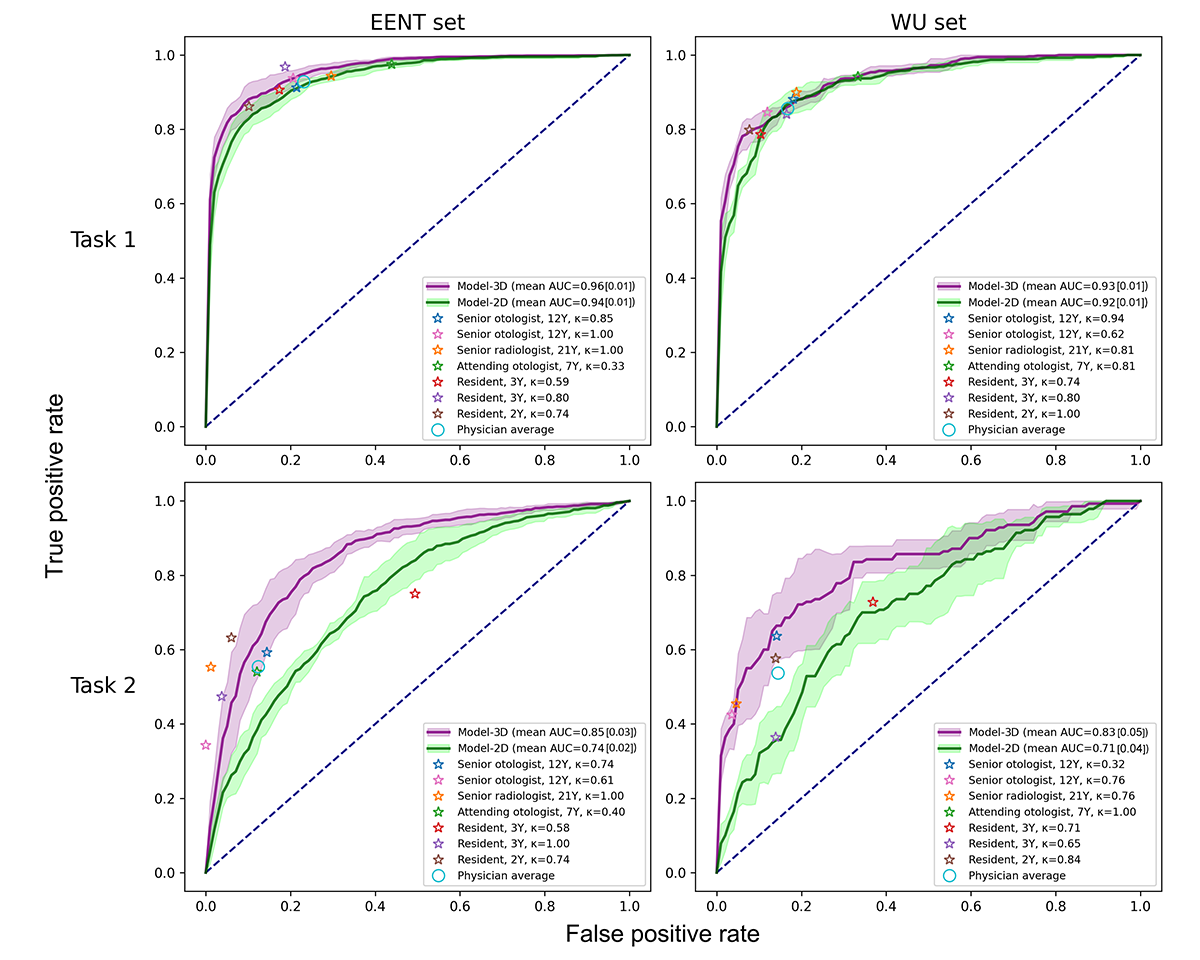
Receiver operating characteristic plots for the benchmark tests. The curve and the shaded area indicate the mean (1 SD) of a model, respectively. Clinical experts are marked by colored asterisks for individual performance and by an open circle for averaged performance. The dotted diagonal line represents a random classifier. AUC: area under the curve; EENT: Eye, Ear, Nose, and Throat Hospital of Fudan University; WU: Wuhan Union Hospital.

Our model exhibited decent performance in identifying pathological changes in the middle ear, achieving a mean accuracy of 87.8%, recall of 85.3%, specificity of 91.3%, and precision of 93.3% on the internal data set (Table 3). It also demonstrated a near-perfect AUROC score of 0.96. These performance metrics remained generally consistent on the external data set, with a comparable AUROC score of 0.93, indicating reasonable generalizability (Figure 4).

**Table 3:** Performance of the baseline 2D and the proposed 3D models.

Task and model			Size (MB)		Data set		Accuracy, mean (SD)		Recall, mean (SD)		Specificity, mean (SD)		Precision, mean (SD)		*F*_1_-score, mean (SD)		AUROC^a^, mean (SD)		*P* value
**1**																			
	3D	14.2		EENT^b^		0.878 (0.017)		0.853 (0.032)		0.913 (0.067)		0.933 (0.045)		0.89 (0.012)		0.00959 (0.00011)		.003	
	2D	274		EENT		0.861 (0.019)		0.845 (0.028)		0.883 (0.052)		0.909 (0.036)		0.875 (0.016)		0.00939 (0.00013)		N/A^c^	
	3D	14.2		WU^d^		0.843 (0.015)		0.756 (0.047)		0.934 (0.021)		0.924 (0.018)		0.83 (0.022)		0.00933 (0.0001)		.05	
	2D	274		WU		0.821 (0.023)		0.744 (0.078)		0.901 (0.046)		0.891 (0.039)		0.808 (0.036)		0.00918 (0.00012)		N/A	
**2**																			
	3D	14.2		EENT		0.783 (0.04)		0.808 (0.025)		0.77 (0.054)		0.652 (0.06)		0.721 (0.042)		0.00853 (0.0003)		<.001	
	2D	274		EENT		0.67 (0.037)		0.716 (0.144)		0.646 (0.119)		0.523 (0.044)		0.596 (0.036)		0.00744 (0.00025)		N/A	
	3D	14.2		WU		0.812 (0.033)		0.614 (0.085)		0.878 (0.031)		0.626 (0.078)		0.618 (0.069)		0.00826 (0.00055)		<.001	
	2D	274		WU		0.676 (0.103)		0.479 (0.224)		0.741 (0.185)		0.41 (0.086)		0.411 (0.096)		0.00714 (0.00049)		N/A	

^a^AUROC: area under the receiver operating characteristic curve.

^b^EENT: Eye, Ear, Nose, and Throat Hospital of Fudan University.

^c^N/A: not applicable.

^d^WU: Wuhan Union Hospital.

### Task 2

This model also demonstrated satisfactory predictive capabilities in differentiating between cholesteatoma and noncholesteatomatous cases. On both data sets, the model managed to correctly identify whether a case involved cholesteatoma in approximately 4 out of 5 instances (with accuracies of 78.3% and 81.3%). Generalizability was further supported by the comparable AUROC scores of 0.85 and 0.83 on the internal and the external data set, respectively (Table 3).

### Ablation Study

This model exhibited a reasonable balance between predictability and efficiency (Table 4). Compared with models 1 and 3, it achieved significantly better performance in both tasks (*P*<.01). In addition, despite having approximately 60% fewer parameters, the proposed model demonstrated equivalent performance to model 2 in both tasks (*P*=.26 and .91, respectively), indicating its enhanced computational efficiency.

**Table 4:** Ablation study on the 3D classification network.

Task and model			Size (MB)		Accuracy, mean (SD)		Recall, mean (SD)		Specificity, mean (SD)		Precision, mean (SD)		*F*_1_-score, mean (SD)		AUROC^a^, mean (SD)		*P* value
**1**																	
	Proposed	14.2		0.878 (0.017)		0.853 (0.032)		0.913 (0.067)		0.933 (0.045)		0.89 (0.012)		0.00959 (0.00011)		N/A^b^	
	Model 1	4.0		0.858 (0.03)		0.827 (0.046)		0.901 (0.058)		0.921 (0.043)		0.87 (0.028)		0.00947 (0.00019)		<.001	
	Model 2	34.5		0.884 (0.014)		0.862 (0.021)		0.914 (0.041)		0.933 (0.03)		0.895 (0.012)		0.00961 (0.00009)		.26	
	Model 3	64.8		0.864 (0.022)		0.851 (0.062)		0.887 (0.074)		0.914 (0.053)		0.878 (0.019)		0.0095 (0.00019)		.003	
**2**																	
	Proposed	14.2		0.783 (4.0)		0.808 (0.025)		0.77 (0.054)		0.652 (0.06)		0.721 (0.042)		0.00853 (0.0003)		N/A	
	Model 1	4.0		0.758 (0.048)		0.712 (0.118)		0.783 (0.065)		0.636 (0.064)		0.668 (0.075)		0.00817 (0.0006)		.006	
	Model 2	34.5		0.782 (0.036)		0.795 (0.071)		0.775 (0.074)		0.659 (0.071)		0.716 (0.032)		0.00862 (0.00031)		.91	
	Model 3	64.8		0.756 (0.056)		0.76 (0.059)		0.754 (0.109)		0.634 (0.088)		0.685 (0.037)		0.00826 (0.000047)		.003	

^a^AUROC: area under the receiver operating characteristic curve.

^b^N/A: not applicable.

### Benchmarks

Compared with the 2D approach, the 3D network demonstrated significantly superior performance in both tasks across data sets (*P*≤.05). In particular, the proposed model exhibited a substantial performance gain in differentiating between cholesteatoma and noncholesteatomata, with an increase of more than 10% in all outcome metrics on both data sets (Table 3).

This model also matched or even surpassed the diagnostic capabilities of human experts in both tasks (Figure 4). It exhibited marginally superior performance compared with human eyes in the first task (*P*=.05) and significantly outperformed them in the visually challenging task 2 (*P*<.001). Post hoc pairwise comparisons revealed that the model excelled over the attending otologist in task 1 and 2 resident fellows in task 2, rivaling all senior clinicians (Table 5). Similar results were shown across the breakdown of data sources, with a notable finding that the model outperformed a senior otologist in task 2 on the EENT subset (Table S4 in Multimedia Appendix 1). Moreover, the proposed model demonstrated perfect consistency, surpassing all human experts who exhibited higher SDs in all outcome metrics and lower scores of intrarater reliability.

**Table 5:** Benchmark performance against human experts.

Task and rater		Accuracy	Recall	Specificity	Precision	*F*_1_-score	Kappa values	*P* value
**1**								
	The 3D model, mean (SD)	0.878 (0.017)	0.853 (0.032)	0.913 (0.067)	0.933 (0.045)	0.89 (0.012)	0.01 (0.00)	N/A^a^
	Expert average, mean (SD)	0.857 (0.022)	0.898 (0.042)	0.804 (0.094)	0.86 (0.05)	0.876 (0.013)	0.0082 (0.0009)	.05
	Senior otologist A: 12 Y^b^	87.3%	89.8%	84.1%	87.9%	88.8%	0.75	.79
	Senior otologist B: 12 Y	85.7%	89.9%	80.4%	85.6%	87.7%	0.92	.49
	Senior radiologist: 21 Y	85.4%	92.4%	76.3%	83.4%	87.7%	0.87	.37
	Attending otologist: 7 Y	81.1%	96.0%	61.9%	76.5%	85.2%	0.73	.002
	Resident A: 3 Y	85.9%	85.5%	86.4%	89.1%	87.2%	0.71	.56
	Resident B: 3 Y	87.4%	91.2%	82.5%	87.1%	89.1%	0.81	.74
	Resident C: 2 Y	86.8%	83.5%	91.2%	92.4%	87.7%	0.92	.96
**2**								
	The 3D model, mean (SD)	0.843 (0.015)	0.756 (0.047)	0.934 (0.021)	0.924 (0.018)	0.83 (0.022)	0.01 (0.00)	N/A
	Expert average, mean (SD)	0.741 (0.052)	0.549 (0.123)	0.865 (0.139)	0.772 (0.135)	0.622 (0.061)	0.0072 (0.0012)	<.001
	Senior otologist A: 12 Y	73.8%	36.7%	98.2%	93.0%	52.6%	0.70	.07
	Senior otologist B: 12 Y	75.8%	60.6%	85.7%	73.3%	66.3%	0.47	.25
	Senior radiologist: 21 Y	79.5%	52.3%	97.0%	91.9%	66.7%	0.86	.82
	Attending otologist: 7 Y	74.5%	55.0%	87.0%	73.2%	62.8%	0.74	.11
	Resident A: 3 Y	63.8%	74.3%	56.9%	52.9%	61.8%	0.67	<.001
	Resident B: 3 Y	72.3%	44.0%	90.9%	76.2%	55.8%	0.77	.02
	Resident C: 2 Y	78.8%	61.5%	89.9%	79.8%	69.4%	0.80	.96

^a^N/A: not applicable.

^b^Y: years of experience in clinical practice.

### Visual Assessment of Heat Maps

**Figure 5:**
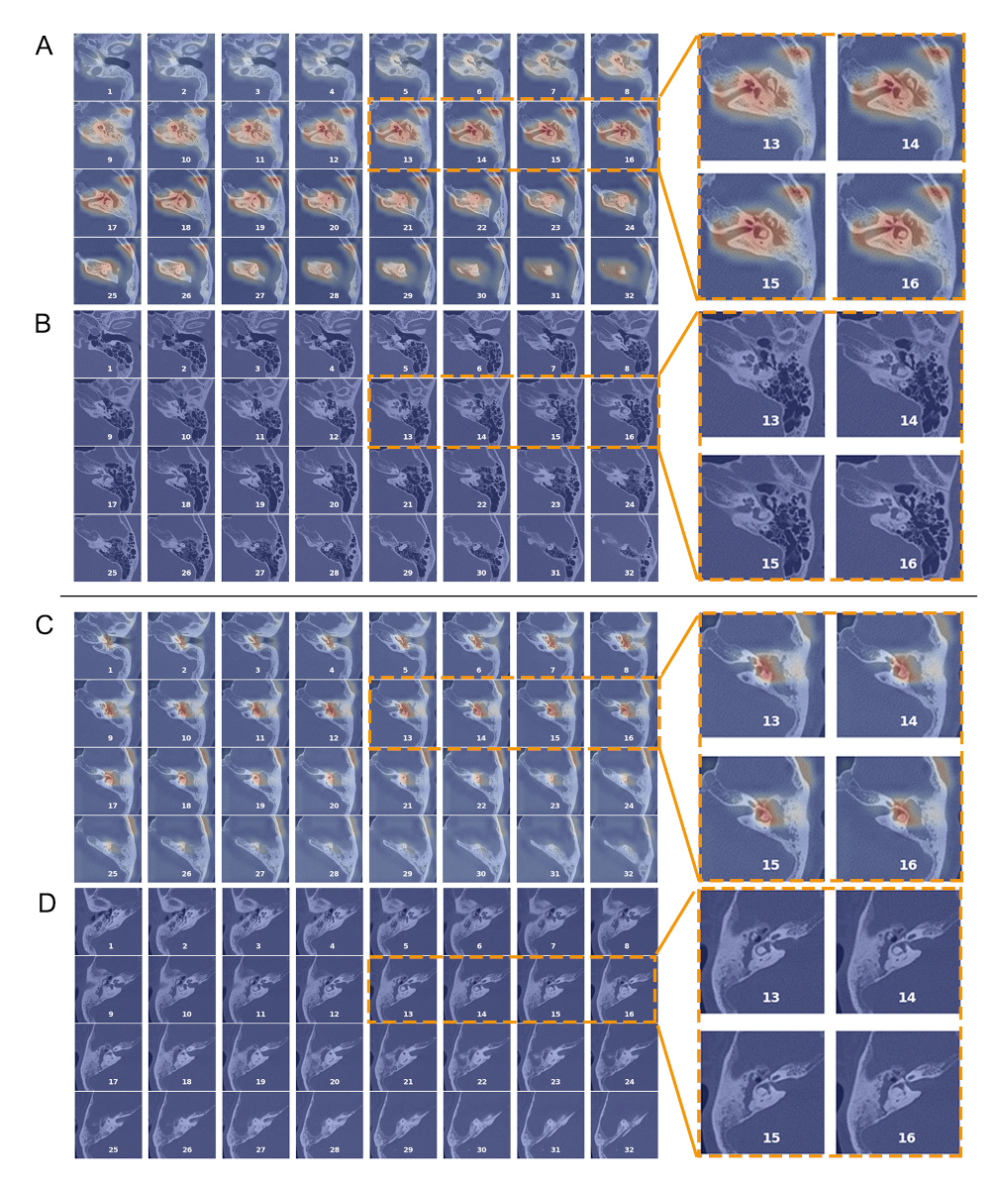
Examples of heat maps. The heat maps, generated in 3D fashion, are superimposed on the original computed tomographic scans and flattened to a series of 2D images for demonstration purpose. (A-B) A pathological and a normal ear, respectively. (C-D) A cholesteatoma and a noncholesteatoma case, respectively. Area marked by hot signals indicate the presence of graphic patterns contributing to a “positive” prediction (ie, a pathological ear in task 1 and a cholesteatoma in task 2).

Heat Maps from both models consistently highlighted the tympanic cavity and mastoid that manifested pathological findings characteristic of the target condition (Figure 5). Specifically, the first model generated a hot signal indicative of soft tissue density in an affected middle ear (Figure 5A), while the signal remained subdued in a normal ear (Figure 5B). Similarly, the second model revealed a distinct hot spot in a cholesteatomatous ear exhibiting the classic patterns of tympanum widening and ossicular destruction [17,32,33] (Figure 5C). In contrast, a case of CSOM showing intact ossicles surrounded by soft tissue shadows in a normal-sized tympanic cavity did not exhibit a corresponding hot spot (Figure 5D). These observations reflect that the AI’s decision-making strategy aligns reasonably well with established human knowledge for both tasks.

### Clinical Use

**Figure 6:**
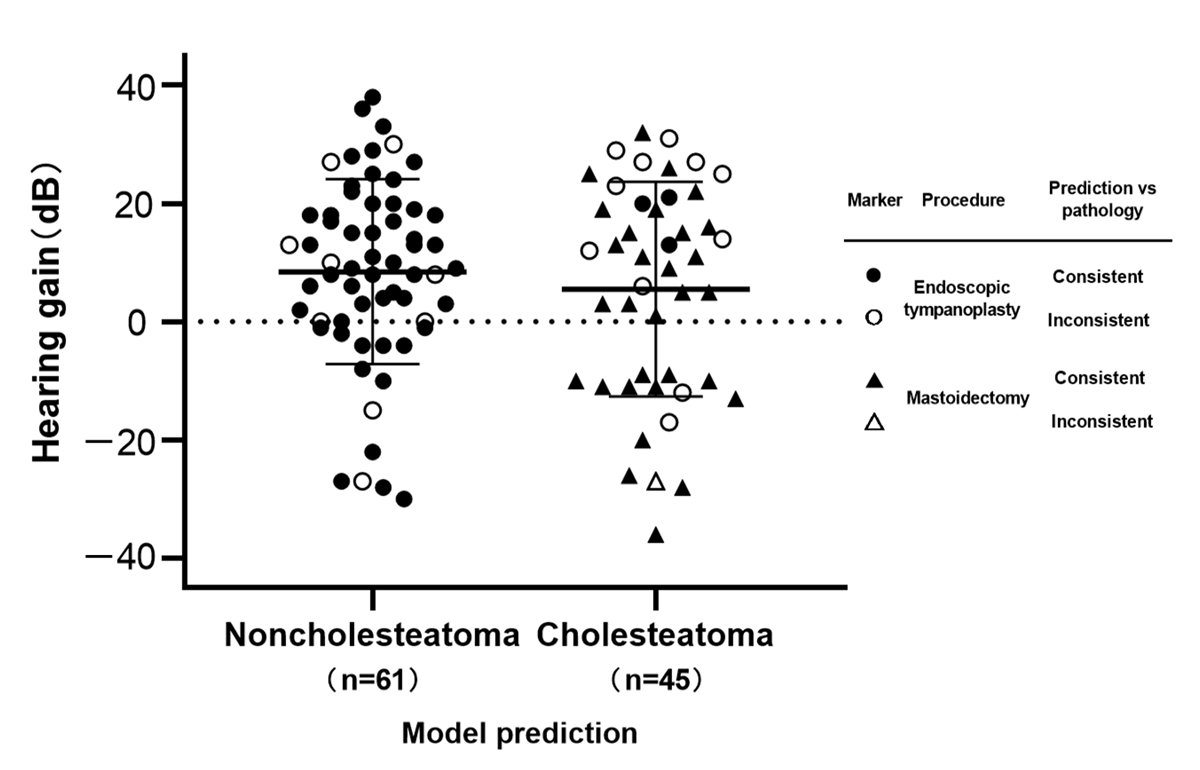
Postoperative hearing gain for the operated ears with available audiometry outcomes (n=106). Data are categorized according to model predictions. Predictions that agree with the pathological results are denoted by close symbols, while open symbols indicate disagreements. Circles and triangles represent the treatment of endoscopic tympanoplasty and mastoidectomy, respectively. The error bars indicate ±1 SD from the mean.

The automatic evaluation system, incorporating the validated 3D model and the heatmap visualization technique, was evaluated for its viability in aiding preoperative assessment in 121 patients with COM (mean age 46.8, SD 16.1 years, 40.5% male). This system achieved an overall accuracy of 81.8% in distinguishing between cholesteatoma and noncholesteatoma cases. Sixty-nine ears were identified as free of cholesteatoma by the model, all of which received minimally invasive tympanoplasty under endoscopy. During the procedure, 9 ears (13.0%) revealed signs of cholesteatoma, and 5 of them required additional bone-grinding technique for complete removal of the mass. Cholesteatoma was initially predicted in 52 ears, with 37 (71.2%) of them undergoing canal-wall-down mastoidectomy. In the remaining 15 ears, the treating surgeons opted for endoscopic tympanoplasty, overriding the conventional technique for the model’s predicted diagnosis. Clinicians reported that the model predictions aligned with their initial judgment or helped with their decision-making in 90.1% (109/121) cases. Postoperative hearing results were obtained in 87.6% (106/121) patients who maintained follow-up. Both groups of ears showed normal recovery, with a mean hearing gain of 8.5 (SD 15.6) and 5.5 (SD 18.1) dB, respectively (Figure 6).

## Discussion

### Principal Results

This study demonstrates the robustness and generalizability of an AI model based on 3D CNN for the detection and differential diagnosis of COM using temporal bone CT scans. This model leverages multidimensional diagnostic information from the middle ear, resulting in a significant performance improvement compared with the traditional 2D approach. The framework exhibits comparable or even superior performances to human experts in otologic tasks with clinical significance and visual challenges, especially for classifying between cholesteatoma and noncholesteatomatous cases. In addition, the novel heatmap technique allows inspection of the AI’s logic for decision-making, thereby enhancing the transparency of this model. The resulting AI system serves to automate summarization of critical radiologic findings and enables efficient evaluation of COM with minimum manual input. It provides tangible benefit in assisting otologists during preoperative assessment and results in favorable clinical outcomes that are comparable with historical results [34-37]. These findings further support the clinical viability and advantages of AI technology, which is expected to improve efficiency, reduce errors, and facilitate precision medicine in health care in the new era of big data.

### Comparison With Prior Work

A few AI models have recently been developed to classify common middle ear conditions, such as CSOM, otitis media with effusion, and cholesteatoma [38-41]. However, these models were primarily based on traditional otoscopic images, which are potentially limited by a narrow field of view and insufficient diagnostic information. Temporal bone CT scans, which are increasingly used in otologic workup by virtue of its accessibility, rich amount of anatomical information, and adequate sensitivity in revealing pathological changes, have also been explored in a limited number of studies [22,42-45]. Although these AI models demonstrated decent AUROC scores (eg, >0.9) in common classification tasks, they were all trained to generate predictions based on 2D single-layer CT scans. A potential drawback is the increased likelihood of missing small or peripheral pathological changes (eg, an attic cholesteatoma) and the resultant false negatives.

Efforts were made in this study to establish a 3D approach to take full advantage of all available anatomical information and achieve a better coverage of the tympanum and the mastoid. Inspection of the extracted ROI suggests that all critical anatomies are visible. Results from the benchmark test indicate that the proposed 3D model outperforms the state-of-the-art 2D approach by a modest performance gain in the detection of COM and by a much larger extent in differentiating between cholesteatoma and noncholesteatoma. This finding has several implications. First, both models are generally adequate in identifying common abnormal patterns from the CT, which are graphically characterized by increased opacification or soft tissue shadows in the middle ear cavity and indicative of pathological conditions in general. This is a relatively simple visual task, during which diagnostic information obtained from a single 2D CT slice is likely sufficient for the purpose and extra findings from other layers provide only minimal contribution to the decision-making. Second, the 3D model has huge advantage over the 2D approach in differentiating cholesteatoma from other types of COM. This task is known to be more visually challenging for humans, often requiring detection of subtle osseous erosions from multiple CT slices, as quite a few pathological changes caused by cholesteatoma are peripheral or noncharacteristic [32,33]. A substantial increase in each outcome measure justifies the advantage of the current 3D model for this task. Moreover, this 3D model has only a simple network structure with a small size (14.5 MB) as opposed to a complex and large-sized 2D network (274 MB), suggesting both higher computational efficiency and performance of the 3D approach. Finally, the AUROC of 0.92-0.94 and accuracy scores of 82.1%-86.1% achieved by the 2D network in this study in detecting COM were equivalent to historical results (0.92%-86%, respectively) in our previous study [22], further indicating the reliability of these findings and potentially the intrinsic limit of using single-layer CT scan for this task. To the best of our knowledge, this is the first study showing quantitative evidence to support the advantage of a 3D CNN model in 2 common otologic tasks based on temporal bone CT scans. It also advances beyond prior retrospective research by showcasing the practicality and benefits of the model in a clinical environment.

### Clinical Implications

Cholesteatoma exhibits distinct histology marked by local invasiveness and a propensity for recurrence. The imperative for successful outcomes necessitates complete removal of the mass, particularly because recurrent cholesteatoma complicates revision surgery [46]. Suspected cases often require a canal-wall-down mastoidectomy to expose the tympanum, resulting in an open cavity and a permanently altered sound conduction pathway [46]. Accumulating evidence suggests that noncholesteatoma may spare from mastoidectomy and benefit from minimally invasive procedures such as endoscopic tympanoplasty [47,48]. Therefore, the current AI system holds potential value for otologists in surgical planning. Ears with a low risk of cholesteatoma, as identified by the model, could potentially be treated by less invasive procedures that retain the integrity of canal wall, leading to reduced procedural time and enhanced recovery [6,7,9,49,50]. This clinical merit is supported by the superior benchmark performance in identifying cholesteatoma and the favorable outcomes observed in the prospective study.

While detecting COM in task 1 involves spotting any pathological patterns on CT, which may not fully capture the differences between models in diagnostic capabilities, the increased visual challenges in identifying cholesteatoma substantiate the advantages of the proposed 3D approach for this task. In this study, the 3D model outperformed junior clinicians and demonstrated equivalent or superior performance to senior experts in identifying cholesteatoma based on CT. Notably, the 3D model achieved outcomes that were on par with or better than those based on human interpretation of MRI, which, despite its higher sensitivity, is a more expensive diagnostic method [22,43,45,51-53]. These findings underscore the 3D model’s potential as a reliable and cost-effective alternative, offering sufficient COM evaluation with CT alone, thereby reducing the need for the pricier MRI.

The findings from the prospective study indicate that the model is efficacious in clinical environments, especially in distinguishing cholesteatoma from noncholesteatoma. Feedback from our clinical team highlights that the system serves as a reliable and streamlined source for a second opinion. Before surgery, the treating physician can rapidly identify essential details such as the lesion’s location and properties, using the model’s diagnostic output, and heatmaps. Concordance between the model’s predictions and the physician’s initial assessment bolsters confidence in surgical planning, thereby streamlining the diagnostic and therapeutic process. In contrast, discrepancies between the model’s results and the physician’s judgment prompt a detailed case reassessment or team consultation, aiding in the validation of a suitable treatment plan or preparing for intraoperative modifications. This process provides timely advisory support for complex cases, encouraging meticulous evaluation by the physician, minimizing errors, and keeping the clinician’s cognitive load in check without compromising their autonomy in decision-making.

It should be noted that even for seasoned otologists and radiologists, who are adept at quickly and accurately reading temporal bone CT scans, a second opinion can add an extra layer of confidence to their assessments. For novice clinicians, who may find the diagnostic process more challenging and time-intensive (Table 5), the model may offer substantial improvements in both the accuracy and the speed of diagnosing and managing COM. This is particularly beneficial for physicians in smaller medical facilities or those early in their careers. Looking ahead, the integration of this model into electronic medical systems or cloud-based servers stands to streamline the provision of immediate second opinions or enable physicians from diverse locations to upload imaging data for dependable diagnostic insights. Such technological progress is poised to advance individualized COM treatments in the big data era, boosting efficiency, reducing costs, and enhancing the quality of patient care.

### Research Insights

Efforts were undertaken in this study to demystify the criticized nontransparency of DL models, characterized by intricate decision-making strategies within multilayer architectures [30,54]. The nonlinear interactions among these components can yield incomprehensible logic and untraceable predictions vulnerable to bias or errors, posing a significant challenge to the widespread application of AI in health care. To address this issue, heatmaps, and specifically, the Gradient-Weighted Class Activation Mapping technique, have been used as a method to inspect AI’s strategy and enhance human interpretation in a parsimonious manner [55-57]. In this study, the strategy learned by our models to focus on the middle ear and mastoid regions appeared reasonable and aligned with human knowledge in interpreting CT for COM, reinforcing the reliability of this framework. These informative heatmaps can aid clinicians in understanding and validating AI predictions for specific cases, or serve as educational tools for training medical students or junior residents in reading temporal bone CT scans. Ultimately, this approach presents a viable solution for developing explainable AI models for clinical tasks.

Overfitting is a common concern with DL models, especially when data are limited or sourced from a single institute. It can lead to poor performance on new data despite promising results on the original data set. Previous DL models were trained on monocentric CT scans with participant counts ranging from 61 to 562. Lack of external validation and small sample sizes may raise concern about potential overfitting of these models [22,42,43]. Several approaches were used in this study to enhance the generalizability of our framework. First, our models underwent cross-validation on a major data set comprising more than 3000 ears, the largest sample size reported to date. Second, these models were evaluated on external data with different patient origins and image properties. Third, several machine learning methods were applied to minimize the risk of models being tuned to the random features, including early termination of training and the use of a dropout function to decrease the interdependency among network nodes [27]. Consistent performance metrics across data sets in both tasks substantiated the generalizability of this framework. Moreover, the region proposal method proved applicable to CT scans from both sources, demonstrating adaptability despite differences in CT scanner, scan settings, and image quality.

### Limitations

**Figure 7:**
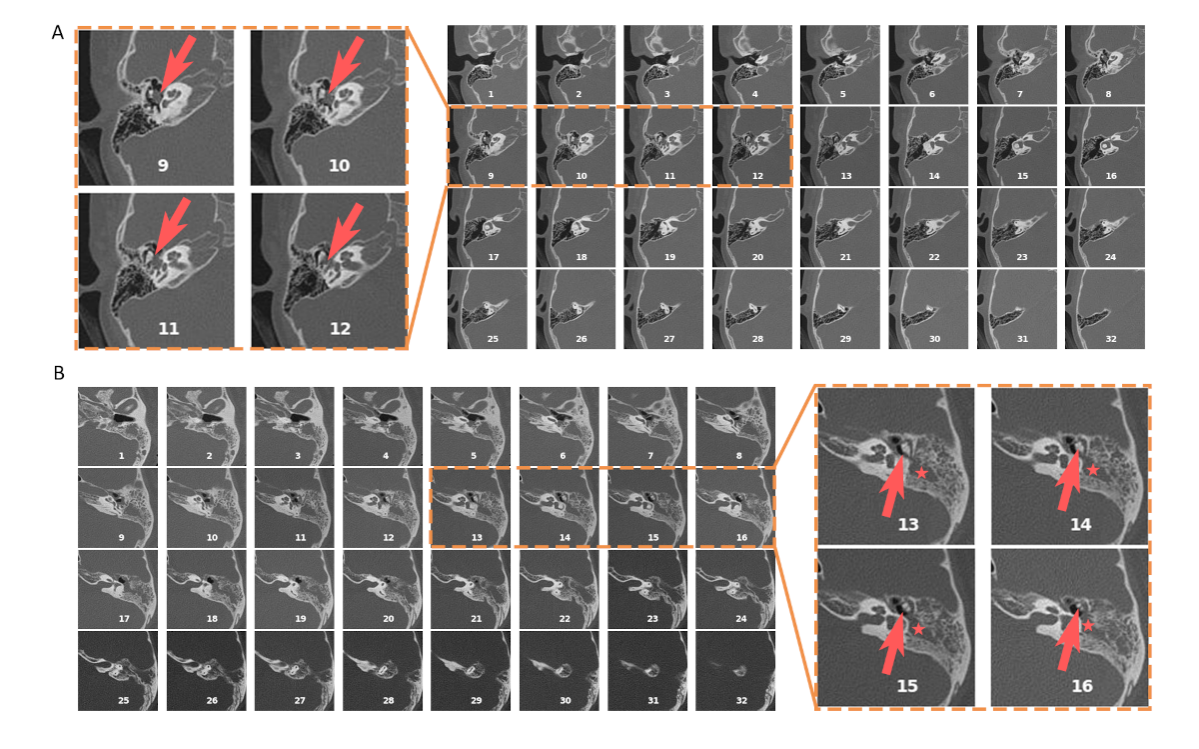
Examples of misclassified cases. (A) A pathological ear showing a small-sized soft tissue density near the ossicles (arrows) with no evident sign of osseous erosion or mastoid opacification. (B) A case of cholesteatoma showing soft tissue density (asterisks) but with a visually intact ossicular chain (arrows) and a normal-sized tympanic cavity.

This study has several limitations. First, although an external data set was obtained from a hospital in a different city, patients in both data sets shared a common racial background. Further validation on data collected from patients with diverse origins may be necessary to ensure the generalizability of these models. Second, the research was constrained to 2 binary classification tasks relevant to COM. Incorporating additional diagnostic tasks, such as assessing the ossicular chain’s integrity and forecasting auditory outcomes, may enrich the diagnostic toolkit. Third, the models were exclusively trained to analyze CT scans, potentially not leveraging AI’s full potential in COM evaluation. Comprehensive diagnostics often involve synthesizing information from patient history, clinical symptoms, ear examinations, audiological testing, otoscopy, and various imaging techniques. Overreliance on CT scans alone may introduce limitations in performance and may not always lead to conclusive diagnoses (Figure 7). Fourth, the ablation study examined a limited array of model alternatives. Despite achieving notable performance through initial model structure refinement, future endeavors should include ongoing optimization of the model architecture and detailed analysis of network component functions to optimize the trade-off between model efficacy and computational demands. In addition, this study did not place extensive emphasis on exploring common ethical issues, such as patient privacy, data security, and human autonomy, which are critical considerations in the clinical application of AI and warrant ongoing attention. Finally, this study reported initial findings from the clinical application of the AI system in a small, prospective cohort without a control group. Although the main objective was to show that the current model is ready for clinical implementation, a thorough assessment of the model’s clinical benefits will be conducted in an upcoming clinical trial with a more rigorous research design.

### Future Research

Future studies will focus on leveraging novel techniques to enhance model performance and evaluate the effectiveness in larger-scale controlled trials. For example, new models will be trained to perform additional tasks, including evaluation of ossicular chain and forecasting postoperative hearing, which may enhance features of the current AI framework. A broader data set will be compiled from hospitals worldwide to assess and refine the generalizability of these models. Moreover, future models will potentially incorporate multiple sources of clinical information with a fusion layer for generating predictions, mimicking human decision-making strategies, and potentially enhancing model robustness. Ongoing efforts will also be made to refine model architectures and to address ethical issues associated with the use of AI in health care. An active learning framework may be established to integrate feedback loops, allowing clinicians to provide input to the model. This approach is expected to support ongoing model enhancement and reinforcement learning based on human feedback. In the next stage, multicenter, prospective human trials will be conducted to assess the practical benefits of implementing these AI models in clinical contexts. The ultimate goal of this research line is to establish a robust AI system that can assist clinicians with reliability, efficiency, and transparency in the evaluation and management of ear diseases.

### Conclusions

This study presents a 3D CNN model trained to detect pathological changes and identify cholesteatoma based on temporal bone CT scans. The model’s performance significantly surpasses the baseline 2D approach, reaching a level comparable with or even exceeding that of human experts in both tasks. The model also exhibits decent generalizability and enhanced comprehensibility through the gradient heatmaps. The resulting AI system allows automatic assessment of COM and shows promising viability in real-word clinical settings. These findings imply the potential of AI as a valuable tool for aiding clinicians in the evaluation of COM. Future research will involve enhancing models with additional source of diagnostic information to perform various clinical tasks and evaluating the benefits of AI models in large-scale controlled trials.

## Appendix

Multimedia Appendix 1 Additional tables.

Multimedia Appendix 2 Source code for model development and validation.

## Abbreviations

AI: artificial intelligence
AUROC: area under the receiver operating characteristic curve
CNN: convolutional neural network
COM: chronic otitis media
CSOM: chronic suppurative otitis media
CT: computed tomography
DL: deep learning
EENT: Eye, Ear, Nose, and Throat Hospital of Fudan University
MRI: magnetic resonance imaging
ROI: region of interest
WU: Wuhan Union Hospital
YOLO: You Only Look Once

## Data Availability

The complete source code and trained models are available in Multimedia Appendices 1 and 2 as well as in a public repository, which can be accessed in GitHub [58]. The automatic evaluation system is available for individual use with a detailed instruction manual and a walk-through tutorial. The data sets generated during this study may be obtained from the corresponding authors upon reasonable request.

## References

[1] Schilder AGM, Chonmaitree T, Cripps AW, Rosenfeld RM, Casselbrant ML, Haggard MP, Venekamp RP (2016). Otitis media. Nat Rev Dis Primers.

[2] World Health Organization (2004). Chronic Suppurative Otitis Media: Burden of Illness and Management Options.

[3] Lustig L, Limb C, Baden R, LaSalvia M (2018). Chronic Otitis Media, Cholesteatoma, and Mastoiditis in Adults.

[4] Takahashi M, Motegi M, Yamamoto K, Yamamoto Y, Kojima H (2022). Endoscopic tympanoplasty type I using interlay technique. J Otolaryngol Head Neck Surg.

[5] Ohki M, Kikuchi S, Tanaka S (2019). Endoscopic type 1 tympanoplasty in chronic otitis media: comparative study with a postauricular microscopic approach. Otolaryngol Head Neck Surg.

[6] Hsu Y, Kuo C, Huang T (2018). A retrospective comparative study of endoscopic and microscopic tympanoplasty. J Otolaryngol Head Neck Surg.

[7] Yang Q, Wang B, Zhang J, Liu H, Xu M, Zhang W (2022). Comparison of endoscopic and microscopic tympanoplasty in patients with chronic otitis media. Eur Arch Otorhinolaryngol.

[8] Tsetsos N, Vlachtsis K, Stavrakas M, Fyrmpas G (2020). Endoscopic versus microscopic ossiculoplasty in chronic otitis media: a systematic review of the literature. Eur Arch Otorhinolaryngol.

[9] Tarabichi M, Ayache S, Nogueira JF, Al Qahtani M, Pothier DD (2013). Endoscopic management of chronic otitis media and tympanoplasty. Otolaryngol Clin North Am.

[10] Watts S, Flood LM, Clifford K (2000). A systematic approach to interpretation of computed tomography scans prior to surgery of middle ear cholesteatoma. J Laryngol Otol.

[11] Selwyn D, Howard J, Cuddihy P (2019). Pre-operative prediction of cholesteatomas from radiology: retrospective cohort study of 106 cases. J Laryngol Otol.

[12] Songu M, Altay C, Onal K, Arslanoglu S, Balci MK, Ucar M, Ciger E, Kopar A (2015). Correlation of computed tomography, echo-planar diffusion-weighted magnetic resonance imaging and surgical outcomes in middle ear cholesteatoma. Acta Otolaryngol.

[13] Mahmutoğlu AS, Celebi I, Sahinoğlu S, Cakmakçi E, Sözen E (2013). Reliability of preoperative multidetector computed tomography scan in patients with chronic otitis media. J Craniofac Surg.

[14] Pandey AK, Bapuraj JR, Gupta AK, Khandelwal N (2009). Is there a role for virtual otoscopy in the preoperative assessment of the ossicular chain in chronic suppurative otitis media? Comparison of HRCT and virtual otoscopy with surgical findings. Eur Radiol.

[15] Chee NW, Tan TY (2001). The value of pre-operative high resolution CT scans in cholesteatoma surgery. Singapore Med J.

[16] Zaman SU, Rangankar V, Muralinath K, Shah V, Pawar R (2022). Temporal bone cholesteatoma: typical findings and evaluation of diagnostic utility on high resolution computed tomography. Cureus.

[17] Gaurano JL, Joharjy IA (2004). Middle ear cholesteatoma: characteristic CT findings in 64 patients. Ann Saudi Med.

[18] Ardila D, Kiraly AP, Bharadwaj S, Choi B, Reicher JJ, Peng L, Tse D, Etemadi M, Ye W, Corrado G, Naidich DP, Shetty S (2019). End-to-end lung cancer screening with three-dimensional deep learning on low-dose chest computed tomography. Nat Med.

[19] Mikhael PG, Wohlwend J, Yala A, Karstens L, Xiang J, Takigami AK, Bourgouin PP, Chan P, Mrah S, Amayri W, Juan Y, Yang C, Wan Y, Lin G, Sequist LV, Fintelmann FJ, Barzilay R (2023). Sybil: a validated deep learning model to predict future lung cancer risk from a single low-dose chest computed tomography. J Clin Oncol.

[20] Yamashita R, Long J, Longacre T, Peng L, Berry G, Martin B, Higgins J, Rubin DL, Shen J (2021). Deep learning model for the prediction of microsatellite instability in colorectal cancer: a diagnostic study. Lancet Oncol.

[21] Li Y, Guo J, Yang P (2022). Developing an image-based deep learning framework for automatic scoring of the pentagon drawing test. J Alzheimers Dis.

[22] Wang Y, Li Y, Cheng Y, He Z, Yang J, Xu J, Chi Z, Chi F, Ren D (2020). Deep learning in automated region proposal and diagnosis of chronic otitis media based on computed tomography. Ear Hear.

[23] Sundgaard JV, Harte J, Bray P, Laugesen S, Kamide Y, Tanaka C, Paulsen RR, Christensen AN (2021). Deep metric learning for otitis media classification. Med Image Anal.

[24] Watson DS, Krutzinna J, Bruce IN, Griffiths CE, McInnes IB, Barnes MR, Floridi L (2019). Clinical applications of machine learning algorithms: beyond the black box. BMJ.

[25] Castelvecchi D (2016). Can we open the black box of AI?. Nature.

[26] Redmon J, Divvala S, Girshick R, Farhadi A (2016). You only look once: unified, real-time object detection.

[27] Srivastava N, Hinton G, Krizhevsky A, Sutskever I, Salakhutdinov R (2014). Dropout: a simple way to prevent neural networks from overfitting. J machine Learn Res.

[28] Kingma DP, Ba J (2014). Adam: a method for stochastic optimization. arXiv.

[29] Tseng C, Lai M, Wu C, Yuan S, Ding Y (2016). Comparison of the efficacy of endoscopic tympanoplasty and microscopic tympanoplasty: a systematic review and meta‐analysis. Laryngoscope.

[30] Selvaraju RR, Cogswell M, Das A, Vedantam R, Parikh D, Batra D (2017). Grad-cam: visual explanations from deep networks via gradient-based localization.

[31] Python Core Team Python: a dynamic, open source programming language. Python Software Foundation.

[32] Baráth K, Huber AM, Stämpfli P, Varga Z, Kollias S (2011). Neuroradiology of cholesteatomas. AJNR Am J Neuroradiol.

[33] Gulati M, Gupta S, Prakash A, Garg A, Dixit R (2019). HRCT imaging of acquired cholesteatoma: a pictorial review. Insights Imaging.

[34] Daneshi A, Daneshvar A, Asghari A, Farhadi M, Mohebbi S, Mohseni M (2020). Endoscopic versus microscopic cartilage myringoplasty in chronic otitis media. Iran J Otorhinolaryngol.

[35] Prasad SC, Melia CL, Medina M, Vincenti V, Bacciu A, Bacciu S, Pasanisi E (2014). Long-term surgical and functional outcomes of the intact canal wall technique for middle ear cholesteatoma in the paediatric population. Acta Otorhinolaryngol Ital.

[36] Wood CB, O’Connell BP, Lowery AC, Bennett ML, Wanna GB (2019). Hearing outcomes following type 3 tympanoplasty with stapes columella grafting in canal wall down mastoidectomy. Ann Otol Rhinol Laryngol.

[37] Chamoli P, Singh CV, Radia S, Shah AK (2018). Functional and anatomical outcome of inside out technique for cholesteatoma surgery. Am J Otolaryngol.

[38] Wu Z, Lin Z, Li L, Pan H, Chen G, Fu Y, Qiu Q (2020). Deep learning for classification of pediatric otitis media. Laryngoscope.

[39] Pichichero ME (2021). Can machine learning and AI replace otoscopy for diagnosis of otitis media?. Pediatrics.

[40] Tseng CC, Lim V, Jyung RW (2023). Use of artificial intelligence for the diagnosis of cholesteatoma. Laryngoscope Invest Otolaryngol.

[41] Livingstone D, Chau J (2020). Otoscopic diagnosis using computer vision: an automated machine learning approach. Laryngoscope.

[42] Duan B, Guo Z, Pan L, Xu Z, Chen W (2020). Temporal bone CT-based deep learning models for differential diagnosis of primary ciliary dyskinesia related otitis media and simple otitis media with effusion. Am J Transl Res.

[43] Eroğlu O, Eroğlu Y, Yıldırım M, Karlıdag T, Çınar A, Akyiğit A, Kaygusuz I, Yıldırım H, Keleş E, Yalçın Ş (2022). Is it useful to use computerized tomography image-based artificial intelligence modelling in the differential diagnosis of chronic otitis media with and without cholesteatoma?. Am J Otolaryngol.

[44] Khosravi M, Jabbari Moghaddam Y, Esmaeili M, Keshtkar A, Jalili J, Tayefi Nasrabadi H (2022). Classification of mastoid air cells by CT scan images using deep learning method. J Big Data.

[45] Wang Z, Song J, Su R, Hou M, Qi M, Zhang J, Wu X (2022). Structure-aware deep learning for chronic middle ear disease. Expert Syst Appl.

[46] Tomlin J, Chang D, McCutcheon B, Harris J (2013). Surgical technique and recurrence in cholesteatoma: a meta-analysis. Audiol Neurotol.

[47] Lee S, Lee DY, Seo Y, Kim YH (2019). Can endoscopic tympanoplasty be a good alternative to microscopic tympanoplasty? a systematic review and meta-analysis. Clin Exp Otorhinolaryngol.

[48] Trinidade A, Page JC, Dornhoffer JL (2016). Therapeutic mastoidectomy in the management of noncholesteatomatous chronic otitis media. Otolaryngol Head Neck Surg.

[49] Wu L, Liu Q, Gao B, Huang S, Yang N (2022). Comparison of endoscopic and microscopic management of attic cholesteatoma: a randomized controlled trial. Am J Otolaryngol.

[50] Toulouie S, Block‐Wheeler NR, Rivero A (2022). Postoperative pain after endoscopic vs microscopic otologic surgery: a systematic review and meta-analysis. Otolaryngol Head Neck Surg.

[51] Profant M, Sláviková K, Kabátová Z, Slezák P, Waczulíková I (2012). Predictive validity of MRI in detecting and following cholesteatoma. Eur Arch Otorhinolaryngol.

[52] Lin M, Sha Y, Sheng Y, Chen W (2022). Accuracy of 2D blade turbo gradient- and spin-echo diffusion weighted imaging for the diagnosis of primary middle ear cholesteatoma. Otol Neurotol.

[53] Sharifian H, Taheri E, Borghei P, Shakiba M, Jalali AH, Roshanfekr M, Firouznia K (2012). Diagnostic accuracy of non‐echo‐planar diffusion‐weighted MRI versus other MRI sequences in cholesteatoma. J Med Imaging Radiat Oncol.

[54] Montavon G, Lapuschkin S, Binder A, Samek W, Müller K (2017). Explaining nonlinear classification decisions with deep Taylor decomposition. Pattern Recognit.

[55] Panwar H, Gupta PK, Siddiqui MK, Morales-Menendez R, Bhardwaj P, Singh V (2020). A deep learning and grad-CAM based color visualization approach for fast detection of Covid-19 cases using chest X-ray and CT-scan images. Chaos Solitons Fractals.

[56] Cheng C, Ho T, Lee T, Chang C, Chou C, Chen C, Chung I, Liao C (2019). Application of a deep learning algorithm for detection and visualization of hip fractures on plain pelvic radiographs. Eur Radiol.

[57] He T, Guo J, Chen N, Xu X, Wang Z, Fu K, Liu L, Yi Z (2020). MediMLP: using grad-CAM to extract crucial variables for lung cancer postoperative complication prediction. IEEE J Biomed Health Inform.

[58] huntlylee / 3D-Otitis-Media. GitHub.

