# Supplementary material for: A 3D and Explainable Artificial Intelligence Model for Evaluation of Chronic Otitis Media Based on Temporal Bone Computed Tomography: Model Development, Validation, and Clinical Application
Source: J Med Internet Res. 2024 Aug 8;26:e51706. doi: 10.2196/51706 (PMC11342006; doi:10.2196/51706)
Supplement: Multimedia Appendix 1 [file jmir_v26i1e51706_app1.docx]

**Table S1.** Summary of Model 1 architecture

| Block | Kernel | Kernel settings |
| --- | --- | --- |
| Input | Input |  |
| Convolution 1 | Conv3D | (3,3,3,64) |
|  | MaxPooling3D | (2,2,2) |
|  | BatchNormalization |  |
| Convolution 2 | Conv3D | (3,3,3,64) |
|  | MaxPooling3D | (2,2,2) |
|  | BatchNormalization |  |
| Convolution 3 | Conv3D | (3,3,3,128) |
|  | MaxPooling3D | (2,2,2) |
|  | BatchNormalization |  |
|  | GlobalAveragePooling3D |  |
| Dense 1 | Fully connected | 64 |
|  | Dropout | 0.3 |
| Output | Fully connected | 2 |

Conv3D: three-dimensional convolutional layer; MaxPooling3D: three-dimensional max pooling layer; BatchNormalization: batch normalization layer; GlobalAveragePooling3D: layer performing global average pooling for three-dimensional data.

**Table S2.** Summary of Model 2 architecture

| Block | Kernel | Kernel settings |
| --- | --- | --- |
| Input | Input |  |
| Convolution 1 | Conv3D | (3,3,3,64) |
|  | MaxPooling3D | (2,2,2) |
|  | BatchNormalization |  |
| Convolution 2 | Conv3D | (3,3,3,64) |
|  | MaxPooling3D | (2,2,2) |
|  | BatchNormalization |  |
| Convolution 3 | Conv3D | (3,3,3,128) |
|  | MaxPooling3D | (2,2,2) |
|  | BatchNormalization |  |
| Convolution 4 | Conv3D | (3,3,3,256) |
|  | MaxPooling3D | (2,2,2) |
|  | BatchNormalization |  |
| Convolution 5 | Conv3D | (3,3,3,256) |
|  | MaxPooling3D | (2,2,2) |
|  | BatchNormalization |  |
|  | GlobalAveragePooling3D |  |
| Dense 1 | Fully connected | 64 |
|  | Dropout | 0.3 |
| Output | Fully connected | 2 |

Conv3D: three-dimensional convolutional layer; MaxPooling3D: three-dimensional max pooling layer; BatchNormalization: batch normalization layer; GlobalAveragePooling3D: layer performing global average pooling for three-dimensional data.

**Table S3.** Summary of Model 3 architecture

| Block | Kernel | Kernel settings |
| --- | --- | --- |
| Input | Input |  |
| Convolution 1 | Conv3D | (5,5,5,64) |
|  | MaxPooling3D | (2,2,2) |
|  | BatchNormalization |  |
| Convolution 2 | Conv3D | (5,5,5,64) |
|  | MaxPooling3D | (2,2,2) |
|  | BatchNormalization |  |
| Convolution 3 | Conv3D | (5,5,5,128) |
|  | MaxPooling3D | (2,2,2) |
|  | BatchNormalization |  |
| Convolution 4 | Conv3D | (5,5,5,256) |
|  | MaxPooling3D | (2,2,2) |
|  | BatchNormalization |  |
|  | GlobalAveragePooling3D |  |
| Dense 1 | Fully connected | 64 |
|  | Dropout | 0.3 |
| Output | Fully connected | 2 |

Conv3D: three-dimensional convolutional layer; MaxPooling3D: three-dimensional max pooling layer; BatchNormalization: batch normalization layer; GlobalAveragePooling3D: layer performing global average pooling for three-dimensional data.

**Table S4.** Detailed breakdown of human performance by dataset

| Task | Dataset | Clinician | Accuracy | Recall | Specificity | Precision | F1 Score | Kappa | *P* |
| --- | --- | --- | --- | --- | --- | --- | --- | --- | --- |
| 1 | EENT | Senior otologist A - 12Y | 88.2% | 93.7% | 79.4% | 88.1% | 90.8% | 1.00 | .094 |
|  |  | Senior otologist B - 12Y | 86.4% | 91.2% | 78.6% | 87.3% | 89.2% | 0.85 |  |
|  |  | Senior radiologist - 21Y | 85.2% | 94.3% | 70.4% | 83.8% | 88.8% | 1.00 |  |
|  |  | Attending otologist - 7Y | 81.7% | 97.5% | 56.1% | 78.3% | 86.8% | 0.33 |  |
|  |  | Resident A - 3Y | 87.5% | 90.6% | 82.7% | 89.4% | 90.0% | 0.59 |  |
|  |  | Resident B - 3Y | 90.8% | 96.8% | 81.3% | 89.3% | 92.9% | 0.80 |  |
|  |  | Resident C - 2Y | 87.5% | 86.2% | 89.8% | 93.2% | 89.5% | 0.74 |  |
|  | WU | Senior otologist A - 12Y | 86.3% | 84.6% | 88.0% | 87.6% | 86.1% | 0.63 | .749 |
|  |  | Senior otologist B - 12Y | 85.0% | 88.1% | 81.9% | 83.2% | 85.6% | 0.94 |  |
|  |  | Senior radiologist - 21Y | 85.6% | 89.9% | 81.2% | 82.9% | 86.3% | 0.81 |  |
|  |  | Attending otologist - 7Y | 80.5% | 94.1% | 66.7% | 74.2% | 83.0% | 0.81 |  |
|  |  | Resident A - 3Y | 84.1% | 78.6% | 89.7% | 88.5% | 83.3% | 0.74 |  |
|  |  | Resident B - 3Y | 83.8% | 84.0% | 83.5% | 84.0% | 84.0% | 0.80 |  |
|  |  | Resident C - 2Y | 86.0% | 79.8% | 92.3% | 91.3% | 85.2% | 1.00 |  |
| 2 | EENT | Senior otologist A - 12Y | 68.4% | 34.2% | 100.0% | 100.0% | 51.0% | 0.61 | **.01** |
|  |  | Senior otologist B - 12Y | 73.0% | 59.2% | 85.5% | 78.9% | 67.7% | 0.74 | .15 |
|  |  | Senior radiologist - 21Y | 78.0% | 55.3% | 98.8% | 97.7% | 70.6% | 1.00 | .93 |
|  |  | Attending otologist - 7Y | 71.7% | 53.9% | 88.0% | 80.4% | 64.6% | 0.40 | .08 |
|  |  | Resident A - 3Y | 62.3% | 75.0% | 50.6% | 58.2% | 65.5% | 0.58 | **<.001** |
|  |  | Resident B - 3Y | 72.3% | 47.4% | 96.2% | 92.3% | 62.6% | 1.00 | .11 |
|  |  | Resident C - 2Y | 79.2% | 63.2% | 94.0% | 90.6% | 74.4% | 0.74 | .77 |
|  | WU | Senior otologist A - 12Y | 81.2% | 42.4% | 96.4% | 82.4% | 56.0% | 0.76 | .99 |
|  |  | Senior otologist B - 12Y | 79.7% | 63.6% | 85.9% | 63.6% | 63.6% | 0.32 | .70 |
|  |  | Senior radiologist - 21Y | 81.5% | 45.5% | 95.3% | 78.9% | 57.7% | 0.76 | .95 |
|  |  | Attending otologist - 7Y | 78.2% | 57.6% | 86.0% | 61.3% | 59.4% | 1.00 | .46 |
|  |  | Resident A - 3Y | 65.8% | 72.7% | 63.1% | 43.6% | 54.5% | 0.71 | **.001** |
|  |  | Resident B - 3Y | 72.3% | 36.4% | 86.0% | 50.0% | 42.1% | 0.65 | **.045** |
|  |  | Resident C - 2Y | 78.2% | 57.6% | 86.0% | 61.3% | 59.4% | 0.84 | .46 |
